# Including cannabinoids in the treatment of painful schwannomatosis

**DOI:** 10.1002/brb3.1011

**Published:** 2018-05-29

**Authors:** Vittorio Iorno, Anna Roberto, Laura Brigitta Colantonio, Laura Landi, Oscar Corli

**Affiliations:** ^1^ Centre for Pain Medicine M. TIENGO Fondazione IRCCS Cà Granda Ospedale Maggiore Policlinico Milan Italy; ^2^ Pain and Palliative Care Research Unit Oncology Department IRCCS Istituto di Ricerche Farmacologiche Mario Negri Milan Italy; ^3^ Anestesia e Rianimazione Pediatrica IRCCS Fondazione Cà Granda Ospedale Maggiore Policlinico Milan Italy

## Abstract

A 47‐year‐old man, affected by Schwannomatosis, presented a very severe pain (10/10, NRS) with paroxysmal shooting episodes, allodynia, paresthesia, and dysesthesia; in parallel, the patient had lost weight (from 70 to 49 kg) and experienced fatigue and deep depression. The previous pain prescription, including opioids and antineutopathic drugs, was fully ineffective. We progressively substituted this therapy with 15 drops, 3 times/daily, of THC/CBD in a concentration ratio 5:1, equal to 15 mg of active substance each time, reaching improvement in pain intensity (6/10) and in several other aspects as mood and quality of life

Schwannomatosis is a rare form of neurofibromatosis affecting one in approximately 40,000 individuals. It is a clinical condition represented by the development of schwannomas, benign encapsulated tumors originating from Schwann cells. Schwannomas preferentially affect the spine (74%) and peripheral nerves (89%).

The distinctive hallmark of Schwannomatosis is intractable and debilitating pain (Gonzalvo et al., 2011; Lu‐Emerson & Plotkin, 2009), reported by about two‐thirds of patients (Merker, Esparza, Smith, Stemmer‐Rachamimov, & Plotkin, 2012). Pain is not strictly linked to tumor growth or mechanical nerve compression. There is growing evidence that Schwann cells contribute to pain by secreting a number of trophic and inflammatory substances including tumor necrosis factor‐α (TNF‐α), that sensitize nociceptors (Campana et al., 2006; Lattanzi et al., 2015; Watkins & Maier, 2003). In addition, TNF‐α leads to inflammatory pain such as neuritis (Romero‐Sandoval & Eisenach, 2007). Nerve growth factor (NGF) is also expressed by Schwann cells promoting pain and long‐lasting hyperalgesia (Lewin, Ritter, & Mendell, 1993; Mills et al., 2013; Rukwied et al., 2010; Thompson, Dray, McCarson, Krause, & Urban, 1995). It is interesting that localized hyperalgesia is attenuated by a transient receptor potential vanilloid 1 (TRPV1) antagonist, suggesting a role of this receptor (Mills et al., 2013) as a potential target of pain treatment.

We describe the medical history of a 47‐year‐old man with Schwannomatosis who accessed our neurology department in November 2016.

The onset in 2011 featured signs and symptoms of neuropathic pain, such as paroxysmal shooting pain in the forearms, legs, and inguinal area, together with superficial mechanical allodynia, paresthesia, and dysesthesia in the same areas. Neurologic and imaging examinations (MRI, EMG, ENG, needle biopsy) revealed multiple neurinomas of unknown cause.

The patient described his pain as ranging from a tingling or needle pricking sensation to that of a classic jolting shock or as if someone were tightly pinching a nerve, making it impossible for him to extend his limbs.

The pain prescriptions (pregabalin 150 mg qd, carbamazepine 400 mg, amitriptyline 35 mg, clonazepam drops 0.5‐0.7 mg before bedtime) had a poor efficacy.

Ten months later, a new series of diagnostic tests (contrast MRI, EMG, ENG, biopsy) lead to the diagnosis of Schwannomatosis with sensory and motor neuropathy.

In June 2013, 6 masses were surgically removed from the right femoral nerve, inguinal and right axillary regions. The nodules were located on the nerve sheaths like beads on a rosary having round, oval or rice grain shapes. After surgery, there was a pain intensity reduction from 8/10 to 6/10 on 0‐10 numerical rating scale (NRS). Dexamethasone (8 mg qd) was added, with initially positive results, but soon interrupted because of hyperarousal. In the following months, the pain became severe again and the quality of life was seriously compromised. At the same time, the patient lost weight (from 70 to 49 kg), experienced sleeping disorders and profound weakness that made him unable to walk and work and fell into a deep depression. His thinness highlighted the rosary shaped neurinomas at the right forearm. In July 2016, a new surgical removal of neurinomas did not improve pain, that reached 10/10.

In November 2016 MRI revealed an increase in number and volume of the preexisting masses and a new pelvic formation, not eligible for surgical treatment.

At that time, the patient came to our attention and his clinical picture was characterized by severe pain (9/10), sudden painful jolts that compromised quality of sleep and walking, with a constant need of a wheelchair. Severe fatigue and full loss of social and professional life appeared.

The pain therapy consisted of pregabalin 1,200 mg/qd, carbamazepine 400 mg, amitriptyline 70 mg, clonazepam 2,5 mg, tramadol 50 mg up to four times qd, but had a very poor efficacy.

The pain therapy unit was consulted and a new therapy schedule was initiated including a high‐protein diet of 4,000 Kcal qd and motor rehabilitation. Pain treatment was changed. Pregabalin was interrupted and the following drugs were administered daily: oxycodone/naloxone 30 + 15 mg; paracetamol 3,000 mg; vitamin B1 300 mg; vitamin D/colecalciferol 35 g; oil solution, as sublingual administration, containing tetra‐hydro‐cannabinol (THC) 19% = 5 g + cannabidiol (CBD) crystal 99% = 1 g (10 sublingual drops, equivalent to 10 mg of active substance, 3 times/daily, after increased until 15 drops equal to 15 mg, 3 times/daily for achieve a better state of well‐being); duloxetine 120 mg; clonazepam drops (4,5 mg). This last treatment has been ongoing for about a year and it is still ongoing.

The patient had an overall improvement in pain scores (6/10), motor activity, quality of sleep, social relations and mood. Progressively the fatigue was reduced and the patient could walk on his own.

Afterward, the daily treatment was newly modified: duloxetine, clonazepam, paracetamol and oxycodone/naloxone daily doses were halved, while THC/CBD was increased to 15 mg q8 hr. Adverse events due to the cannabinoids were never observed.

Due to a progressive clinical improvement, the opioids have been currently suspended, and duloxetine and clonazepam reduced by another half. The patient gained weight (65 kg) and returned to walk and work.

The more impressive aspect of this clinical case is the reduction of pain after introducing cannabinoids in the therapeutic schedule, especially after the complete failure of the previous pain therapy, including opioids and antineuropathic drugs. In parallel, many aspects of the patient’s quality of life improved too. An interpretative key can originate from the recent finding that cannabidiol exerts a pharmacological action on TRPV1 (Transient Receptor Potential Vanilloid 1). This receptor is a nonselective nociceptive cation channel, especially facilitating the entry of Ca^2+^ into the C‐ and Aδ‐type sensory nerve endings. This influx leads to the release of proinflammatory cytokines, PGs and NGF. The first two families are strictly connected with the inflammatory pain, while NGF produces a long‐lasting increase in mechanical and thermal sensitivity (Mills et al. 2013). NGF causes pain by directly acting on nociceptors (Pezet & McMahon, 2006) and hypersensitivity by increasing TRPV1 expression. Cannabidiol causes TRPV1 dephosphorylation and dose‐dependently desensitizes them rapidly.

For certain, CBD differs from all the other analgesic drugs considering its pharmacological target and mechanism of action. The simultaneous improvement of mood, quality of life and sleep could rather be attributed to the psychotropic properties of THC, able to induce energizing effects and whet appetite.

The choice of giving CBD and THC together has been useful in the case described and can be suggested whenever other therapy schedules fail to relieve pain and other symptoms in patients with Schwannomatosis. This choice could be based on the synergy of the two compounds, the first mainly active on neuropathy and the second on the inflammatory component and modulation of the THC metabolism, limiting its psychoactive effects.

## CONFLICT OF INTEREST

None of the authors has any conflict of interest to disclose.
